# mRNA Expression of *SMPD1* Encoding Acid Sphingomyelinase Decreases upon Antidepressant Treatment

**DOI:** 10.3390/ijms22115700

**Published:** 2021-05-27

**Authors:** Cosima Rhein, Iulia Zoicas, Lena M. Marx, Stefanie Zeitler, Tobias Hepp, Claudia von Zimmermann, Christiane Mühle, Tanja Richter-Schmidinger, Bernd Lenz, Yesim Erim, Martin Reichel, Erich Gulbins, Johannes Kornhuber

**Affiliations:** 1 Department of Psychiatry and Psychotherapy, Friedrich-Alexander-Universität Erlangen-Nürnberg (FAU), Schwabachanlage 6, D-91054 Erlangen, Germany; iulia.zoicas@uk-erlangen.de (I.Z.); Lena.M.Marx@web.de (L.M.M.); Stefanie.Zeitler@web.de (S.Z.); claudia.von.zimmermann@uk-erlangen.de (C.v.Z.); christiane.muehle@uk-erlangen.de (C.M.); tanja.richter-schmidinger@uk-erlangen.de (T.R.-S.); bernd.lenz@zi-mannheim.de (B.L.); martin.reichel@charite.de (M.R.); johannes.kornhuber@uk-erlangen.de (J.K.); 2 Department of Psychosomatic Medicine and Psychotherapy, Friedrich-Alexander-Universität Erlangen-Nürnberg (FAU), D-91054 Erlangen, Germany; Tobias.Hepp@uk-erlangen.de (T.H.); yesim.erim@uk-erlangen.de (Y.E.); 3 Institute of Medical Informatics, Biometry and Epidemiology, Friedrich-Alexander-Universität Erlangen-Nürnberg (FAU), D-91054 Erlangen, Germany; 4 Department of Addictive Behavior and Addiction Medicine, Central Institute of Mental Health (CIMH), Medical Faculty Mannheim, Heidelberg University, D-68159 Mannheim, Germany; 5 Department of Molecular Biology, University Hospital, University of Duisburg-Essen, D-45147 Essen, Germany; erich.gulbins@uk-essen.de

**Keywords:** acid sphingomyelinase, *SMPD1*, major depressive disorder, antidepressants, amitriptyline, fluoxetine, FIASMA, biomarker

## Abstract

Major depressive disorder (MDD) is a severe psychiatric condition with key symptoms of low mood and lack of motivation, joy, and pleasure. Recently, the acid sphingomyelinase (ASM)/ceramide system has been implicated in the pathogenesis of MDD. ASM is a lysosomal glycoprotein that catalyzes the hydrolysis of sphingomyelin, an abundant component of membranes, into the bioactive sphingolipid ceramide, which impacts signaling pathways. ASM activity is inhibited by several common antidepressant drugs. Human and murine studies have confirmed that increased ASM activity and ceramide levels are correlated with MDD. To define a molecular marker for treatment monitoring, we investigated the mRNA expression of *SMPD1*, which encodes ASM, in primary cell culture models, a mouse study, and a human study with untreated MDD patients before and after antidepressive treatment. Our cell culture study showed that a common antidepressant inhibited ASM activity at the enzymatic level and also at the transcriptional level. In a genetically modified mouse line with depressive-like behavior, *Smpd1* mRNA expression in dorsal hippocampal tissue was significantly decreased after treatment with a common antidepressant. The large human study showed that *SMPD1* mRNA expression in untreated MDD patients decreased significantly after antidepressive treatment. This translational study shows that *SMPD1* mRNA expression could serve as a molecular marker for treatment and adherence monitoring of MDD.

## 1. Introduction

Major depressive disorder (MDD) is a severe psychiatric condition with a complex pathophysiology. Key psychological symptoms are persistent low mood and a lack of motivation, joy, and pleasure. Physical symptoms include changes in appetite or weight, sleep disturbances, and unexplained pain. Social problems typically arise and result in difficulties in work, home and family life. With a lifetime prevalence of more than 10% [1], MDD is a very common disorder, but its pathogenesis is still not fully elucidated. The diathesis-stress model suggests that the interaction between a biologic predisposition and psychosocial stress triggers pathologic symptoms. In particular, the nature of the disease implies dysregulation of the cytokine, neurotransmitter, and hormonal systems [2,3,4,5]. Recently, the acid sphingomyelinase (ASM)/ceramide system has been implicated in the pathogenesis of MDD. ASM (human and murine Asm, EC 3.1.4.12) is a lysosomal glycoprotein that catalyzes the hydrolysis of sphingomyelin, an abundant component of membranes, into ceramide and phosphorylcholine [6]. Ceramide is a bioactive lipid [7] that affects downstream signaling in the cell and mediates increased apoptotic potential, differentiation or senescence [8,9,10,11]. ASM is mainly located in the lysosome, but can also be secreted [12]. Most notably, several common antidepressant drugs, such as amitriptyline, desipramine, and fluoxetine, inhibit ASM activity, making ASM a very interesting target for psychiatry [13,14,15]. These drugs are suggested to act indirectly via lysosomal trapping [16] and therefore are called ‘functional inhibitors of ASM activity’ (FIASMAs) [17,18]. Murine and human studies have confirmed the subsequent hypothesis that increased ASM activity and ceramide levels are correlated with MDD pathogenesis. In murine studies, dysregulation of sphingolipid metabolism is associated with depressive-like behavior. Transgenic mice overexpressing Asm (Asm-tg) exhibit increased Asm activity and increased hippocampal ceramide levels, resulting in depressive-like behavior [19,20,21]. In a more direct approach, naïve mice that receive infusions of ceramide Cer16:0 into the dorsal hippocampus develop a depressive-like phenotype [22]. Conversely, the elicitation of depressive-like behavior via chronic unpredictable stress [23] results in increased levels of ceramide Cer16:0, Cer16:1, Cer18:1, Cer22:1, and Cer26:1, and in the reduction of several sphingomyelin species in the hippocampus and frontal cortex [24]. Additionally, the administration of corticosterone, which induces depressive-like behavior [25], increases ceramide Cer22:1 levels in the dorsal hippocampus and ceramide Cer20:0, Cer22:1, Cer24:1, Cer26:0, and Cer26:1 levels in the ventral hippocampus [26]. In depressed patients, ASM activity levels in blood cells are significantly increased as compared with a control group and correlate positively with the severity of depressive symptoms [27]. Plasma levels of several ceramides including Cer16:0, Cer18:0, Cer20:0, Cer24:1, and Cer26:1 are increased in patients experiencing a major depressive episode [28], and plasma levels of ceramide species Cer16:0, Cer18:0, Cer20:0, Cer22:0, Cer24:0, and Cer24:1 are also increased in patients with MDD and bipolar disorder [29]. Higher plasma levels of ceramide Cer16:0 and Cer18:0 and sphingomyelin SM18:1 have been associated with higher severity of depression symptoms in patients with coronary artery disease [30]. Conversely, plasma sphingomyelin SM26:1 [31], SM21:0, and SM21:1 [32] levels are decreased in MDD patients, and the SM23:1/SM16:0 ratio is negatively correlated with the severity of depression symptoms [33]. An illustrative overview of the ASM/ceramide system in MDD give several reviews of our group [18,34,35,36].

Our previous analysis of the patterns of ASM splice variant transcripts in blood cells of MDD patients [37] indicated that *SMPD1* transcription could be a suitable diagnostic marker for MDD. In the current study, we focus on the mRNA expression of full-length transcript variant 1 of *SMPD1* (NM_000543.4, termed ASM-1), the only splice form currently known to encode an enzymatically fully active protein [38]. Our results suggest that *SMPD1* transcription is a molecular marker for monitoring MDD treatment.

## 2. Results

### 2.1. Fluoxetine Decreases SMPD1 mRNA Expression in Primary Cell Culture Systems

**Figure 1:**
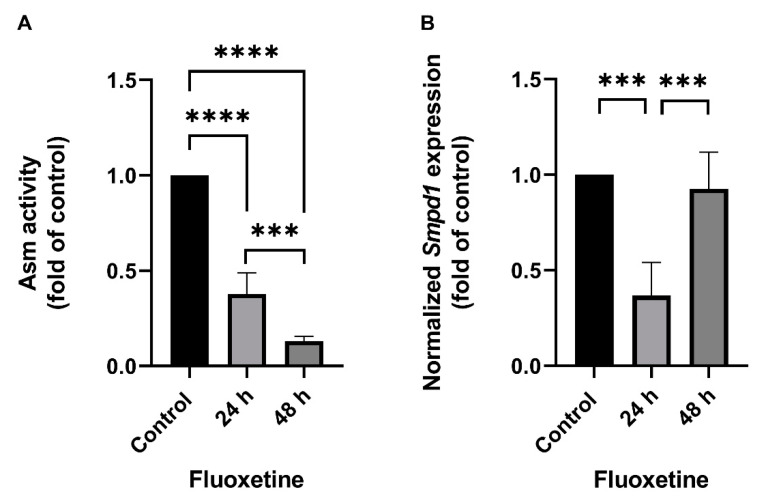

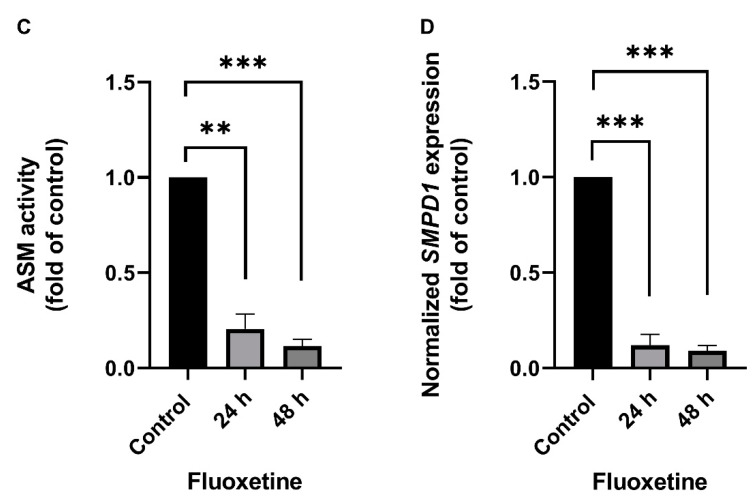
Fluoxetine decreases both ASM activity and *SMPD1* mRNA expression in cultured primary cells. Cultured primary cells were treated a single time with 10 µM fluoxetine and harvested after 24 h or 48 h. (**A**) Asm activity in murine cortical neurons. Fluoxetine inhibited Asm activity at both time points significantly; (**B**) *Smpd1* transcription in murine cortical neurons. Transcription was significantly decreased at 24 h after the single fluoxetine application but was similar to the control at 48 h, N = 5 independent cortex preparations; (**C**) ASM activity in human PBMCs. Fluoxetine inhibited ASM activity at both time points significantly; (**D**) *SMPD1* transcription in human PBMCs. A single stimulation with fluoxetine significantly decreased transcription at both time points. N = 2 independent PBMC preparations, measured in triplicate. Data represent the mean and SD; ** *p* < 0.01, *** *p* < 0.001, **** *p* < 0.0001.

The effect of FIASMA antidepressants on *SMPD1* transcription was investigated in murine primary neuronal cells and human PBMCs. Primary neuronal cells were isolated from the cortices of five wild-type mice, cultured, and stimulated once with 10 µM fluoxetine or PBS as a control condition. Asm activity and *Smpd1* mRNA expression were assessed 24 h and 48 h after stimulation. Asm activity was inhibited after fluoxetine stimulation as compared with controls (Figure 1A, ANOVA, F(2, 12) = 233.8, *p* < 0.0001). The post hoc analysis revealed a significant decrease in Asm activity 24 h (Tukey, *p* < 0.0001) and 48 h (*p* < 0.0001) after stimulation as compared with controls. In addition, the inhibition of Asm activity was stronger at 48 h than at 24 h (*p* < 0.001). The single fluoxetine stimulation also significantly inhibited *Smpd1* transcription as compared with controls (Figure 1B, F(2, 11) = 23.57, *p* = 0.0001). The post hoc analysis revealed that 24 h after stimulation, *Smpd1* transcription was significantly decreased as compared with controls (Tukey, *p* = 0.0001). By contrast, *Smpd1* transcription at 48 h after stimulation was very similar to control levels (*p* = 0.71) and differed significantly from that at 24 h after stimulation (*p* < 0.001).

Human PBMCs were isolated from the full blood of two healthy volunteers by Ficoll density-gradient centrifugation, cultured, and one time stimulated with 10 µM fluoxetine or PBS. ASM activity was inhibited after the single fluoxetine stimulation as compared with controls (Figure 1C, F(2, 3) = 195.0, *p* < 0.001). The post hoc analysis revealed a significant decrease 24 h (Tukey, *p* < 0.01) and 48 h after fluoxetine stimulation as compared with controls (*p* < 0.001). No statistically significant difference was observed between the two time points (*p* = 0.30). *SMPD1* transcription was significantly decreased after the single stimulation as compared with controls (Figure 1D, F(2, 3) = 400.9, *p* < 0.001). The post hoc analysis showed a significant decrease 24 h (Tukey, *p* < 0.001) and 48 h after stimulation (*p* < 0.001). No statistically significant difference was observed between the two time points (*p* = 0.72). Thus, the FIASMA fluoxetine inhibited ASM activity in primary cells and also decreased *SMPD1* mRNA expression after a single stimulation. This effect seems to be reversible, in terms of ASM activity as well as *SMPD1* mRNA expression.

### 2.2. Antidepressant Treatment Decreases Smpd1 mRNA Expression in Dorsal Hippocampal Brain Tissue of Asm-tg Mice

**Figure 2:**
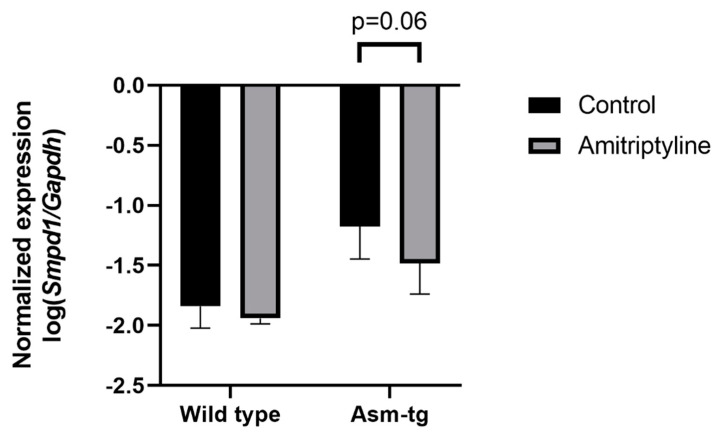
*Smpd1* mRNA expression is decreased in dorsal hippocampal brain tissue of Asm-tg mice after antidepressant treatment. After a daily treatment with amitriptyline for four weeks, *Smpd1* mRNA expression was decreased in Asm-tg mice as compared with controls. N = 5. Data represent the mean and SD.

The transgenic Asm-tg mice used in our study overexpress Asm constitutively, resulting in depressive-like symptoms [19,21]. The genetic strategy of the Asm-tg mouse model implies an increase in *Smpd1* mRNA expression as compared with wild-type mice. To determine the effect of antidepressant treatment on *Smpd1* mRNA expression, we investigated the *Smpd1* mRNA expression profiles of Asm-tg and wild-type mice after a daily treatment for four weeks with the common antidepressant amitriptyline as compared with control groups that received water. We analyzed brain tissue from the dorsal and ventral hippocampus and the frontal cortex. In the dorsal hippocampus, *Smpd1* mRNA expression levels differed significantly among the four experimental groups, i.e., wild-type control (*n* = 6), wild-type amitriptyline-treated (*n* = 5), Asm-tg control (*n* = 5), and Asm-tg amitriptyline-treated (*n* = 5) (2-way ANOVA, genotype effect, F(1, 17) = 38.24, *p* < 0.0001 and treatment effect, F(1, 17) = 5.07, *p* < 0.05, Figure 2). The strong genotype effect confirmed the genetic strategy of the mouse model. Regarding treatment, the post hoc analysis revealed a trend of decreased *Smpd1* mRNA expression in MDD Asm-tg amitriptyline-treated mice as compared with ASM-tg control mice (Sidak, *p* = 0.06). Treatment with amitriptyline did not affect *Smpd1* mRNA expression in wild-type mice (Sidak, *p* = 0.68). The treatment effect observed in Asm-tg mice was specific to the dorsal hippocampus, as these effects could not be detected in the ventral hippocampus and the frontal cortex (data not shown). Thus, *Smpd1* mRNA expression was decreased by FIASMA treatment in vitro and in vivo.

### 2.3. Comparison of SMPD1 mRNA Expression in Blood Cells of Depressed Patients and Healthy Controls

**Figure 3:**
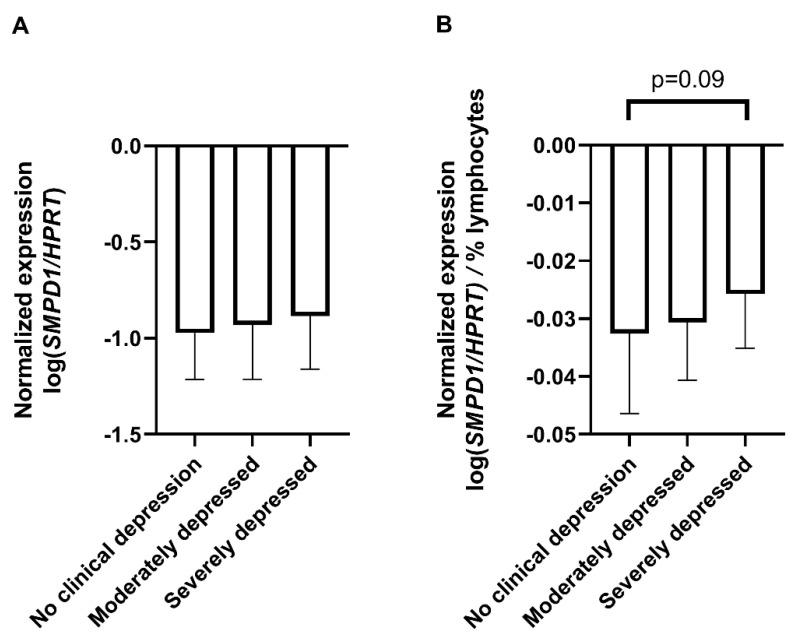
*SMPD1* mRNA expression in blood cells of unmedicated depressed patients and healthy controls. (**A**) No differences between depressed patients and healthy controls. *SMPD1* mRNA expression did not differ between healthy and moderately or severely depressed patients; (**B**) Impact of lymphocyte percentage on *SMPD1* expression differences between depressed patients and healthy controls. When taking the lymphocyte percentage into account, there was a trend in our sample towards an increase in *SMPD1* mRNA expression in severely, but not moderately depressed patients as compared with individuals without clinical depression. Data represent the mean and SD.

To assess the translational aspect of our mouse study, we investigated *SMPD1* mRNA expression in blood cells of untreated patients suffering from MDD and healthy controls. Patients did not receive any antidepressant medication at least two weeks before sampling and the beginning of the treatment. Blood cell expression of *SMPD1* did not differ significantly between depressed patients (*n* = 60) and healthy controls (*n* = 61, ANOVA, F(1, 119) = 1.26, *p* = 0.27). Including the factor sex did not affect results. For a more differentiated analysis [39], the sample was categorized into four groups, according to Hamilton depression scale (HAM-D-17) scores as suggested by clinical practice guideline S3 in psychiatry, i.e., 0–8 points, no clinical depression (*n* = 61); 9–16 points, mild depression (*n* = 0); 17–24 points, moderate depression (*n* = 46); and 25 points and higher, severe depression (*n* = 14). Blood cell expression of *SMPD1* did not differ significantly between depressed patients and healthy controls (ANOVA, F(2, 118) = 0.78, *p* = 0.46, Figure 3A). While analyzing blood parameters of the study sample, we observed a significant difference in the percentage of lymphocytes between the three groups (ANOVA, F(2, 125) = 3.17, *p* < 0.05). The post hoc analysis revealed a higher percentage of lymphocytes in severely depressed patients (36.6% ± 10.1) compared with moderately depressed patients (31.2% ± 6.5, Tukey, *p* < 0.05) and healthy participants (31.6% ± 7.8, Tukey, *p* = 0.06). To rule out a confounding effect of heterogeneity of the sample composition [40], we calculated the *SMPD1* mRNA expression per percentage of lymphocytes. ANOVA revealed a trend towards statistical significance regarding an increased level of *SMPD1* mRNA expression in our sample of depressed patients (F(2, 118) = 2.39, *p* = 0.10). The post hoc analysis revealed a trend towards increased *SMPD1* mRNA expression in severely depressed patients as compared with individuals without clinical depression (*p* = 0.09, Figure 3B).

### 2.4. Antidepressant Treatment Decreases SMPD1 mRNA Expression in Blood Cells of Untreated Depressed Patients

Next, we monitored the changes in *SMPD1* mRNA expression in untreated MDD patients before and after antidepressant treatment. Therapy included pharmacologic treatment and standardized psychotherapeutic interventions for 3 weeks. To evaluate the effect of therapeutic treatment on symptom severity, we compared the Beck Depression Inventory (BDI-II) scores before and after treatment. The MDD patients scored significantly higher on the BDI-II scale before therapy than after 3 weeks of treatment, indicating a significant amelioration of depressive symptoms (*n* = 60, ANOVA with repeated measures, F(1, 59) = 87.2, *p* < 0.001).

**Figure 4:**
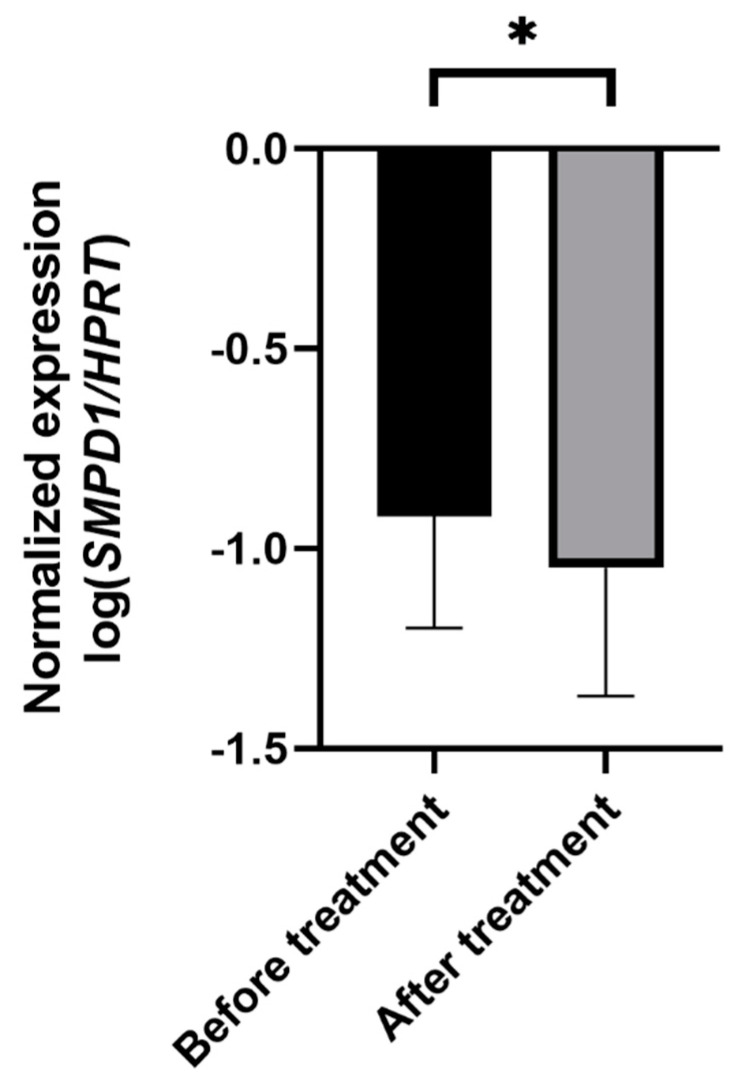
Effects of antidepressant treatment on *SMPD1* mRNA expression. Unmedicated MDD patients displayed significantly higher *SMPD1* mRNA expression before treatment as compared with after treatment. Data represent the mean and SD. * *p* < 0.05.

In depressed patients, *SMPD1* mRNA expression decreased significantly from study inclusion to follow-up (*n* = 52, ANOVA with repeated measures, F(1, 50) = 4.75, *p* < 0.05, Figure 4). No significant difference in treatment effects regarding *SMPD1* mRNA expression was evident between severely (*n* = 11) and moderately (*n* = 41) depressed patients (*p* = 0.99).

To investigate the association between changes in symptom severity and *SMPD1* mRNA expression after therapy, we performed correlative analyses. The changes in symptom severity measured with BDI-II correlated significantly with changes in *SMPD1* mRNA expression after treatment (r = 0.3; *p* < 0.05). 

## 3. Discussion

Our translational study points to a potential role for *SMPD1* mRNA expression analysis in treatment monitoring of MDD, as treatment with common FIASMA-type antidepressants decreased *SMPD1* transcription in vitro and in vivo in mice and humans. Few studies on the role of ASM were performed at the transcriptional level, and our study demonstrates the relevance of *SMPD1* transcription for MDD treatment monitoring.

It is well known that FIASMAs inhibit ASM activity at the enzymatic level in different cell types [18], but most previous studies used only cell lines [14]. Here, we showed that fluoxetine is able to inhibit ASM activity also in primary cells. Then, we analyzed whether FIASMAs might also affect the transcriptional level of *SMPD1.* We investigated both brain and blood primary cells to draw conclusions for those cell types analyzed in our in vivo studies. The FIASMA fluoxetine reduced *SMPD1* transcription in both brain and blood cells under nonpathologic conditions, an important finding with translational implications. However, the effects in brain and blood cells over time differed. In cortical murine neurons, Asm activity and *Smpd1* transcription decreased by approximately 50% 24 h after fluoxetine stimulation. After 48 h, the inhibitory effect on Asm activity was even stronger than that at 24 h, whereas *Smpd1* mRNA expression returned to control levels again. These differential effects suggest that counterregulation of RNA levels restores Asm activity levels. Balanced Asm activity is essential for proper functioning of the cell [41], and it is possible that *Smpd1* mRNA expression is upregulated as soon as cellular Asm activity decreases to a specific level to maintain optimal conditions. However, this regulatory effect was not evident in primary human blood cells; both 24 h and 48 h after fluoxetine stimulation, ASM activity and *SMPD1* mRNA expression were reduced by approximately 80% as compared with control conditions. Thus, the parameters of this regulatory effect could be cell type specific; unlike neuronal cells, which are not a dividing population and depend on cell survival, blood cells do not need to tolerate the treatment. Another potential explanation is differences between human and murine cell metabolism. A recent study in which cells of the murine fibroblast cell line L929 were treated with the direct ASM activity inhibitor ARC39 provides support for a regulatory function of *SMPD1* transcription [42]. After 2 h and 24 h of stimulation with ARC39, residual Asm activity of 5–10% was observed. By contrast, after 2 h of ARC39 stimulation, *Smpd1* mRNA expression was increased two-fold as compared with the control and was equal to control values 24 h after stimulation. Thus, the extreme decrease in Asm activity might result in an increase in mRNA expression to produce more ASM molecules as soon as the inhibitory stimulus ends. This mechanism seems to be highly dependent on the time frame and might vary between cell types and species. The prolonged time to restore *SMPD1* mRNA expression levels in human PBMCs could be helpful to measure treatment effects in blood.

Our murine study provided in vivo confirmation of the effects of FIASMAs on *Smpd1* transcription observed in cell culture, as long-term application of amitriptyline resulted in decreased *Smpd1* mRNA expression levels. This observation is even more remarkable because *Smpd1* transcription is genetically increased in the Asm-tg mouse model. However, FIASMA application was sufficient to decrease this very high level of mRNA expression. It is also possible that this effect is due to changes in mRNA stability. By contrast, no effect was evident in wild-type mice, which have a relatively low *Smpd1* mRNA expression level. Thus, the effects of FIASMAs on *Smpd1* transcription in vivo appear to be dependent on the basal mRNA expression level or pathologic conditions. Interestingly, the effect of amitriptyline was observed specifically in the dorsal hippocampus, not in the ventral hippocampus or the frontal cortex. This specificity is in line with earlier studies suggesting that the dorsal hippocampus is the most important site for the role of ASM in MDD [19,20,22]. Of note, the dorsal hippocampus is mainly involved in cognitive functions, whereas the ventral part is more related to stress, emotion, and affect. Thus, ASM dysregulation might contribute to MDD symptoms related to cognitive functions.

We further investigated the effect of long-term antidepressive treatment on *SMPD1* transcription observed in our mouse study in a clinical study. As a first attempt, we analyzed whether *SMPD1* transcription could serve as a diagnostic marker for MDD. However, *SMPD1* mRNA expression levels did not differ significantly between healthy controls and patients suffering from MDD, due to the high variability of measured values. This result is in contrast to our earlier study, where we defined the percentage of alternative spliced ASM forms as a differential marker between patients and controls [37]. The results of that previous study pointed to an important diagnostic role for the regularly spliced form ASM-1, but we were unable to confirm *SMPD1* transcription as a diagnostic marker in the present study. Interestingly, analysis of the blood composition of the human study population revealed that lymphocyte percentages differed significantly according to symptom severity. Due to the potential distribution effect on mRNA expression, we included this aspect in our analysis. Indeed, when mRNA expression was calculated per lymphocyte percentage, severely depressed patients showed an increase in *SMPD1* transcription as compared with healthy controls. We collected whole blood for mRNA isolation, but it has been suggested that the highest amount of mRNA is generated by PBMCs and not by granulocytes [43,44]. The majority of cell types in PBMCs are lymphocytes [45]. Thus, including the percentage of lymphocytes in expression analysis seems to be a useful approach. Furthermore, ASM seems to be of high relevance for lymphocyte biology. ASM activity is reportedly higher in isolated lymphocytes than in other blood cell types [46]. In particular, T cells exhibit very high ASM activity as compared with B cells or NK cells (M. Reichel, personal communication). ASM plays an important role in CD4^+^ T-cell signaling [47,48] and regulates regulatory T-cell development [49]. In a murine study, T-cell-specific ASM overexpression resulted in increased T-cell function [50], whereas ASM deficiency seemed to have the opposite effect [51]. The relations between T-cell subpopulations are dysregulated in bipolar disorder [52]. A meta-analysis showed the importance of lymphocyte percentage in MDD, as the ratio of neutrophils to lymphocytes differed between MDD patients and healthy controls and was suggested as a biomarker of inflammatory activation in mood disorders [53]. Thus, lymphocytes seem to play an important role in MDD and ASM biology and should be a focus of future studies. An alternative explanation for the finding of different *SMPD1* mRNA expression in lymphocytes of depressed and healthy individuals could be that the lymphocytes in depressed patients have a slightly different phenotype, maturation, or activation status than those of healthy controls. Such differences in lymphocytes might translate into different levels of expression of ASM in these cells. Treatment with antidepressants might normalize the phenotype and, in turn, *SMPD1* expression.

The effect of long-term antidepressant treatment on *SMPD1* transcription observed in ASM-tg mice with depressive-like behavior was also evident in patients suffering from MDD. A three-week treatment resulted in decreased *SMPD1* transcription levels. Stronger effects might be observed in real-life settings where antidepressants are applied for many months or years. Further studies should monitor *SMPD1* transcription over a longer time course and the effects on sphingolipid levels in PBMCs. Our data suggest that *SMPD1* transcription is a biomarker for treatment monitoring, since the changes in symptom severity were reflected in transcriptional changes. Periodic mRNA expression analysis of patients could be helpful for timely and effective treatment.

An issue that should be addressed in the future is the mechanism of *SMPD1* transcription inhibition by FIASMAs. In contrast to the effect of short-term application of FIASMA on *SMPD1* transcription, long-term application does not seem to have regulatory effects. The current model of ASM activity inhibition suggests that FIASMAs inhibit ASM activity by lysosomal trapping and subsequent proteolysis [16]. If ASM biology is also affected by FIASMAs at the transcriptional level, this model should be expanded.

Limitations of the study are the quite small number of animals in the mouse study and the small number of severely depressed patients in the clinical study. In addition, the types of antidepressant drugs administered in the clinical study were not determined experimentally but were selected according to clinical considerations.

In summary, we demonstrated at three experimental levels, i.e., cell culture, a mouse model, and a clinical study, that FIASMA antidepressants affect *SMPD1* transcription. Our results might be helpful for further research on ASM biology in MDD and could be applied for treatment monitoring in MDD.

## 4. Materials and Methods

### 4.1. Murine Studies

#### 4.1.1. Animal Welfare Declaration

All experiments were performed according to the European Committees Council Directive 2010/63/EU, German law for animal care “TierSchG” 18.05.2006 (BGBl. I S. 1206, 1313), last updated 17 December 2018 (BGBl. I S. 2586), and the German regulation for protection of animals used for scientific purposes “TierSchVersV” 01 August 2013 (BGBl. I S. 3125, 3126), last updated 31 August 2015 (BGBl. I S. 1474).

#### 4.1.2. Murine Primary Neuronal Cell Culture

Brains of wild-type C57BL/6N mice were dissected within 24 h after birth (postnatal days 0–1), and the cortices were isolated, transferred into HBSS-containing tubes, and treated with PDD solution (HBSS -/-, 0.01% papain (*w*/*v*) (Worthington Biochemical Corporation, Lakewood, NJ, USA), 0.1% (*w*/*v*) dispase II (Hoffmann-La Roche AG, Basel, Switzerland), 0.01% (*w*/*v*) DNase I (Worthington Biochemical Corporation), 12.4 mM MgSO_4_) [54], followed by mechanical trituration. A total of 2 × 10^5^ cells per well were seeded in an NBA-media mix (Neurobasal A, 1% (*v*/*v*) GlutaMax, 2% (*v*/*v*) B27 50x, 1% (*v*/*v*) sodium pyruvate, 1% (*v*/*v*) antibiotic/antimycotic (all from Gibco/Thermo Scientific, Schwerte, Germany)) in 12-well plates coated with poly-L-lysine (2.5 mg/mL poly-L-lysine (Sigma-Aldrich, Munich, Germany) in 150 mM borate buffer pH 8.4). Cells were cultured for one week at 37 °C in a 5% CO_2_ humidified atmosphere and treated as indicated with 10 µM fluoxetine [15].

RNA isolation was performed with the Purelink RNA Kit from Thermo Scientific (Schwerte, Germany), according to the manufacturer’s protocol. RNA qualities and concentrations were assessed using a NanoDrop ND-1000 UV-Vis spectrophotometer (Peqlab, Erlangen, Germany). Five hundred nanograms of RNA were reverse transcribed into cDNA using the Quanta cDNA Kit (Gaithersburg, MD, USA), according to the manufacturer’s protocol.

#### 4.1.3. Mouse Specimens and Treatment

Male Asm-tg mice conditionally overexpressing Asm and male wild-type littermates (each *n* = 5–6, 10 weeks of age) from the F1 generation were used in this study (approved by the Committee on the Ethics of Animal Experiments of the Government of Unterfranken, permit number 55.2–2532.1–27/11). The animals were individually housed and treated with amitriptyline (amitriptyline hydrochloride, A8404, Sigma-Aldrich, Munich, Germany) in drinking water at a dose of 180 mg/l or with water as a control condition for four weeks, as previously described [19,21]. All mice consumed a similar amount of amitriptyline.

#### 4.1.4. RNA Isolation and cDNA Synthesis of Murine Tissue

Total RNA was isolated from pieces of mouse brain tissue (<30 mg) using a TissueLyser LT bead mill (Qiagen, Hilden, Germany) and peqGOLD Trifast reagent (Peqlab, Erlangen, Germany), according to the manufacturers’ instructions, followed by RNA purification using the Purelink RNA Kit (Thermo Scientific, Schwerte, Germany). Five hundred nanograms of RNA were transformed into cDNA using the Quanta cDNA Kit (Gaithersburg, MD, USA), according to the manufacturer’s protocol.

#### 4.1.5. Quantitative PCR

Quantitative PCR was performed using a LightCycler 480 real-time PCR system (Roche, Mannheim, Germany) in SYBR green format. The qPCR analyses were performed in a reaction volume of 10 µL containing 5 μL of FastStart Essential DNA Green Master Mix (Roche, Germany), primers at a final concentration of 1 µM each (Operon, Ebersberg, Germany), and 2.5 µL of diluted cDNA. The temperature profile used was 95 °C for 5 min followed by 40 cycles of amplification (95 °C for 10 s, 60 °C for 20 s, and 72 °C for 30 s). The following primers were used for murine material: *Smpd1* (forward: 5′-TGCTGAGAATCGAGGAGACA-3′, reverse: 5′-GACCGGCCAGAGTGTTTTC-3′), reference gene *Gapdh* (forward: 5′-AGGTCGGTGTGAACGGATTTG-3′, reverse: 5′-TGTAGACCATGTAGTTGAGGTCA-3′) [55]. Threshold cycles (Ct) were determined with the second-derivative maximum method, and relative mRNA expression levels were calculated with the 2−^ΔΔCt^ method [56] using LightCycler 480 software (release 1.5.0); mRNA expression levels were transformed to a logarithmic scale (log10) where indicated.

### 4.2. Human Studies

#### 4.2.1. Ethics Statement

The collection of blood samples was approved by the Ethics Committee of Friedrich-Alexander-University Erlangen-Nürnberg (FAU, ID 148_13 B, 17 July 2013) and conducted in concordance with the Declaration of Helsinki. Written informed consent was obtained from all participants.

#### 4.2.2. Human PBMC Primary Cell Culture

Nine milliliters of blood were drawn in EDTA-containing tubes from healthy male volunteers taking part in the FLIP-MD study. The study was approved by the Ethics Committee of Friedrich-Alexander University Erlangen-Nürnberg (194_16 B). Peripheral blood mononuclear cells (PBMCs) were isolated by Ficoll-density gradient (Biochrom/Merck, Darmstadt, Germany) centrifugation (10 min, 1.000 *g*) using Leucosep tubes (Greiner Bio-one, Kremsmünster, Austria). PBMCs (1 × 10^6^ per well) were seeded in RPMI-1640 media containing FCS (Thermo Scientific, Schwerte, Germany) in 12-well plates, cultured for two days at 37 °C in a 5% CO_2_ humidified atmosphere and treated as indicated.

#### 4.2.3. Study Samples

We analyzed human samples that were collected for the CeraBiDe (“Ceramide-associated Biomarkers in Depression”) study [57,58,59,60]. Here, we included MDD patients who were currently experiencing depression, were unmedicated, and had not received medication for at least 2 weeks (*n* = 60) and a healthy control group (*n* = 61). After admission to the hospital, the unmedicated patients received treatment as usual, including an adjustment of psychotropic drug administration. Unmedicated patients were treated with the following psychotropic drugs: bupropion, escitalopram, lorazepam, mirtazapine, opipramol, pipamperone, risperidone, sertraline, valproic acid, venlafaxine. The most often used drugs were mirtazapine, sertraline and escitalopram. The inclusion criteria were age 18–75 years and BMI 18.5–35.0 kg/m^2^. The exclusion criteria were severe physical illness, autoimmune disorders, pregnancy, breastfeeding, and use of anti-inflammatory drugs or corticosteroids within the last 7 days. All participants were screened using the structured clinical interview for DSM-IV (SKID-I). To assess the severity of depression in self-evaluation, the 21-item Beck Depression Inventory II (BDI-II, [61]) was applied as a psychometric scale; symptom severity according to clinician-administered ratings was assessed using the Hamilton depression rating scale (HAM-D-17). The sample was categorized into four groups according to the Hamilton depression scale (HAM-D-17) scores as suggested by clinical practice guideline S3 in psychiatry: 0–8 points, no clinical depression (*n* = 61); 9–16 points, mild depression (*n* = 0); 17–24 points, moderate depression (*n* = 46); and 25 points and higher, severe depression (*n* = 14). Whole blood collection for RNA analysis (collected after overnight fasting in the morning) and psychometric assessment were performed at baseline and after a mean of 22.8 days (SD = 6.8) of treatment. For eight of the patients, there was no RNA available at the follow-up time point.

#### 4.2.4. RNA Isolation and cDNA Synthesis

Fasting blood samples for RNA extraction were drawn in PAXgene TM Blood RNA tubes (Qiagen, Hilden, Germany) and stored at −80 °C; RNA was isolated according to the manufacturer’s instructions. The concentration of RNA was determined photometrically using a NanoDrop ND-1000 UV-Vis spectrophotometer (Peqlab, Erlangen, Germany). Five hundred nanograms of RNA were used in a 20 μL reverse transcription reaction using the Quanta cDNA Kit (Gaithersburg, MD, USA) to synthesize cDNA.

#### 4.2.5. Quantitative PCR

Quantitative PCR was performed using a LightCycler 480 real-time PCR system (Roche, Mannheim, Germany) in SYBR green format. qPCR analyses were performed in a reaction volume of 10 µL containing 5 μL of FastStart Essential DNA Green Master Mix (Roche, Germany), primers at a final concentration of 1 µM each (Operon, Ebersberg, Germany), and 2.5 µL of diluted cDNA. The temperature profile used was 95 °C for 5 min followed by 40 cycles of amplification (95 °C for 10 s, 60 °C for 20 s, 72 °C for 30 s). The following primers were used for human material: *SMPD1* (transcript variant ASM-1; forward: 5′-CCTCAGAATTGGGGGGTTCTATGC-3′, reverse: 5′-CACACGGTAACCAGGATTAAGG-3′), reference gene *HPRT* (5′-TCCGCCTCCTCCTCTGCTC-3′, reverse: 5′-GAATAAACACCCTTTCCAAATCCTCA-3′) [38]. Threshold cycles (Ct) were determined with the second-derivative maximum method, and relative mRNA expression levels were calculated with the 2−^ΔΔCt^ method [56] using LightCycler 480 software (release 1.5.0). mRNA expression levels were transformed to a logarithmic scale (log10) where indicated.

### 4.3. Enzymatic Activity Assay

The enzymatic activity of ASM in cells was investigated, as previously described [62]. Briefly, 1 µg of protein, determined using a bicinchoninic acid kit (Sigma, Germany), was incubated with 0.58 µM *N*-(4,4-difluoro-5,7-dimethyl-4-bora-3a,4a-diaza-s-indacene-3-dodecanoyl)-sphingosylphosphocholine (BODIPY^®^ FL C_12_-sphingomyelin, D-7711, Life Technologies, Darmstadt, Germany) in a 50 µL reaction buffer (50 mM sodium acetate pH 5.0, 0.3 M NaCl, and 0.2% NP-40) for 2 h at 37 °C; after incubation, 3 µL of the reaction mixture was spotted on a silica gel 60 plate (Macherey-Nagel, Düren, Germany), and the spots of ceramide and sphingomyelin were separated by thin layer chromatography using 99% ethyl acetate/1% acetic acid (*v*/*v*) as a solvent. The intensities of the BODIPY-conjugated ceramide and sphingomyelin fractions were determined using a Typhoon Trio scanner (GE Healthcare, München, Germany) and quantified with QuantityOne software (BioRad, München, Germany).

### 4.4. Statistical Analysis

Statistical analysis was performed using IBM SPSS Statistics version 21 (SPSS Inc., Chicago, IL, USA) and GraphPad Prism for Windows, Version 4.01 (GraphPad Software, LaJolla, CA, USA). Statistical significance was determined using (2-way) ANOVA and post hoc analysis with respective corrections or ANOVA with repeated measures. Correlations were calculated using Pearson’s r. A two-sided *p*-value < 0.05 was considered to indicate statistical significance. In addition, effect sizes with two-sided *p*-values < 0.1 are referred to as trends present in the available sample but should not be generalized. All the results are presented as the mean value ± standard deviation (SD).

## Data Availability

The datasets generated during and/or analyzed during the current study are available from the corresponding author on reasonable request.
